# Functional analysis of the group A streptococcal *luxS*/AI-2 system in metabolism, adaptation to stress and interaction with host cells

**DOI:** 10.1186/1471-2180-8-188

**Published:** 2008-10-30

**Authors:** Maria Siller, Rajendra P Janapatla, Zaid A Pirzada, Christine Hassler, Daniela Zinkl, Emmanuelle Charpentier

**Affiliations:** 1 Max F. Perutz Laboratories, University of Vienna, Department of Microbiology and Immunobiology, 1030 Vienna, Austria; 2 The Laboratory for Molecular Infection Medicine Sweden (MIMS), Umeå University, S-90187 Umeå, Sweden

## Abstract

**Background:**

The *luxS*/AI-2 signaling pathway has been reported to interfere with important physiological and pathogenic functions in a variety of bacteria. In the present study, we investigated the functional role of the streptococcal *luxS*/AI-2 system in metabolism and diverse aspects of pathogenicity including the adaptation of the organism to stress conditions using two serotypes of *Streptococcus pyogenes*, M1 and M19.

**Results:**

Exposing wild-type and isogenic *luxS*-deficient strains to sulfur-limited media suggested a limited role for *luxS *in streptococcal activated methyl cycle metabolism. Interestingly, loss of *luxS *led to an increased acid tolerance in both serotypes. Accordingly, *luxS *expression and AI-2 production were reduced at lower pH, thus linking the *luxS*/AI-2 system to stress adaptation in *S. pyogenes*. *luxS *expression and AI-2 production also decreased when cells were grown in RPMI medium supplemented with 10% serum, considered to be a host environment-mimicking medium. Furthermore, interaction analysis with epithelial cells and macrophages showed a clear advantage of the *luxS*-deficient mutants to be internalized and survive intracellularly in the host cells compared to the wild-type parents. In addition, our data revealed that *luxS *influences the expression of two virulence-associated factors, the *fasX *regulatory RNA and the virulence gene *sibA *(*psp*).

**Conclusion:**

Here, we suggest that the group A streptococcal *luxS*/AI-2 system is not only involved in the regulation of virulence factor expression but in addition low level of *luxS *expression seems to provide an advantage for bacterial survival in conditions that can be encountered during infections.

## Background

Bacterial cell density-dependent signaling, also termed "quorum sensing", is used by bacteria to collectively modulate gene expression in response to changes in the population density [1-6]. Although signaling can be achieved through a variety of regulatory mechanisms, all systems described to date involve the production, secretion and detection of extracellular low-molecular-weight signaling molecules called "autoinducers" [1,3,7,8]. Due to the specificity of their respective sensors, recognition of acylated homoserine lactones (AHLs) (gram-negative bacteria) and peptide autoinducers (gram-positive bacteria) is restricted for communication within the same species. More recently, a novel quorum sensing system involving a furanone-like signaling molecule termed "autoinducer 2" (AI-2) was described to regulate bioluminescence in *Vibrio harveyi *[9,10]. AI-2 is synthesized by the *luxS *gene product, which has been identified in the genome of over 55 gram-negative and gram-positive bacterial species [1,11,12]. Since culture supernatants of several bacterial species had a complementary effect on *luxS*-deficient *V. harveyi*, AI-2 has been proposed to function as a "universal" signaling molecule for interspecies communication [1,9,10]. In addition to controlling bioluminescence, recent studies show that the *luxS*/AI-2 signaling is involved in the regulation of pathogenicity in several organisms [5,12].

Apart from its role in quorum sensing, the enzyme LuxS is tightly coupled to the *S*-adenosylmethionine (SAM) utilization pathway [11-13]. SAM is an essential donor of methyl groups for DNA, RNA and other methylation reactions. Its utilization by methyl transferases yields *S*-adenosylhomocysteine (SAH), which is toxic for the cells and is eliminated through hydrolysis by the nucleosidase Pfs to produce *S*-ribosylhomocysteine (SRH) and adenine. Finally, LuxS cleaves SRH into homocysteine and 4,5-dihydroxy-2,3-pentanedione (DPD). DPD spontaneously forms the cyclic pro-AI-2 molecule, which in *V. harveyi *reacts with borate to form a stable cyclic furanosyl borate diester [1,11,13].

Knowledge about quorum-sensing systems in the gram-positive human pathogen *Streptococcus pyogenes *is rather limited. *S. pyogenes *(Group A Streptococcus, GAS) is responsible for a broad range of diseases including mild illnesses such as pharyngitis, impetigo and scarlet fever and more severe invasive infections such as necrotizing fasciitis, streptococcal toxic shock syndrome and post-infectious rheumatic fever [14,15]. In GAS, like in other bacteria, pathogenicity is multifactorial and requires the coordinated temporal regulation of virulence factor expression in response to changing environmental conditions and cell population density [14,16,17]. Studies have confirmed that expression of several GAS virulence genes is temporal and dependent on growth phase [16-20]. Furthermore, a number of specific regulators (e.g. two-component systems and response regulators) modulate virulence gene expression in a growth phase dependent manner [16,21-24]. Based on similarities with previously described peptide signaling regulons of *S. pneumoniae *(*com *system) and *S. aureus *(*agr *system), two putative quorum-sensing systems, *sil *and *fasBCA*, have been reported in GAS [21,25]. Although no signaling peptide could be identified within the *fasBCA *locus, the putative pheromone peptide SilCR from the *sil *operon was shown to regulate DNA uptake and the ability of GAS to cause invasive infection [21,25]. In addition, the observation of a *luxS *homologue in the GAS genome suggested that quorum sensing via the *luxS*/AI-2 signaling could have a relevant function in the pathogenesis of this organism [26,27]. Two recent studies report a role of *luxS*/AI-2 in the regulation of virulence gene expression in M3 and M6 serotypes [26,27]. However, the role of the *luxS*/AI-2 in the AMC-related metabolism and adaptation to stress conditions in GAS remains unknown.

Here, we were interested in investigating further the function of the *luxS*/AI-2 system in GAS serotypes M1 and M19. Expression of *luxS *in connection with production of AI-2 like activity was analyzed. The metabolic role of *luxS *in the activated methyl cycle (AMC) in GAS was examined. Wild-type and isogenic *luxS*-deficient strains were compared in regard to their adaptation to diverse growth and stress conditions as well as diverse aspects of pathogenicity including interaction with epithelial cells and macrophages. In summary, our data suggest an important function of the *luxS*/AI-2 system in survival and growth of GAS under conditions that are relevant during infections.

## Results

### Construction of *luxS*-deficient mutants

As reported previously, GAS possesses a *luxS *homolog (coding sequence: 483 bp), the predicted translational product of which shares 36% identity with the LuxS protein from *V. harveyi *[26]. Analysis of the *luxS *coding sequences in clinical isolates of two different serotypes, RDN29 (M1 type) and RDN02 (M19 type), revealed 100% amino acid sequence identity. In a previous study, a GAS strain of serotype M6 (JRS4) was shown to contain in its genome the insertion element, IS *1239*, inserted 111 bp upstream of the *luxS *translational start codon [26]. This insertion was assumed to have an influence on *luxS *expression [26]. Further analysis of the sequence up to 300 bp upstream of the *luxS *coding sequence in RDN29 and RDN02 did not reveal the presence of IS *1239 *(data not shown). To analyze the function of the *luxS*/AI-2 system in GAS, we deleted the *luxS *gene in RDN29 and RDN02, thus creating EC478 and RDN306, respectively (Table 1).

**Table 1:** Bacterial strains and plasmids used in this study

Strain/plasmid	Relevant characteristics	Source
*Streptococcus pyogenes*		
RDN29	M1 type	ATCC 700294
RDN02	M19 type	Lab strain collection
EC478	Isogenic *luxS*-deficient mutant of RDN29	This study
RDN306	Isogenic *luxS*-deficient mutant of RDN02	This study
EC460	RDN29 (pEC82)	This study
EC489	EC478 (pEC82)	This study
EC490	EC478 (pEC83)	This study
EC480	RDN02 (pEC82)	This study
EC481	RDN306 (pEC82)	This study
EC482	RDN306 (pEC83)	This study
		
*Escherichia coli*		
DH5α	Host for cloning; AI-2 deficient (contains a frame-shift mutation in *luxS*)	Lab strain collection
TOP10	Host for cloning	Invitrogen
EC467	DH5α (pEC82)	This study
EC464	DH5α (pEC83)	This study
		
*Vibrio harveyi*		
BB170	AI-1 sensor deficient, AI-2 sensor positive	[9]
Plasmids		
pAT21	Ap^r ^Km^r^; pBR322Ω 1.5-kb pJH1 *Cla*I (*aphIII*)	[61]
pUC19	ColE1ori, Amp^r^, *lacZ*	New England Biolabs
pEC131	pUC19Ω*luxS*up-*aphIII*-*luxS*down	This study
pEC82	*repDEG*-pAMβ1, *ermAM/B*, ColE1ori	This study
pEC83	pEC82ΩP*luxS*-*luxS*-TT	This study

### Temporal *luxS *expression and production of AI-2 like activity

**Figure 1:**
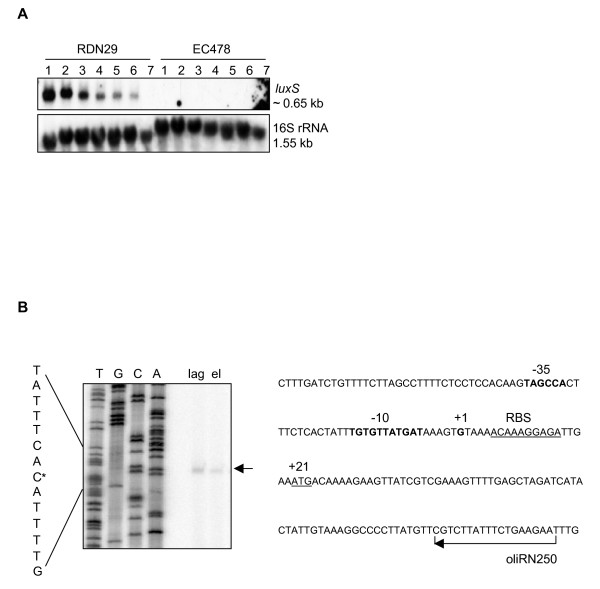
**Analysis of the *luxS *transcript in *S. pyogenes*.** (A) Temporal analysis of *luxS *expression. Northern blot analysis of total RNA isolated from RDN29 (wild-type M1 strain) and EC478 (isogenic *luxS*-deficient mutant) cultures at (1) lag, (2) early-logarithmic, (3) mid-logarithmic, (4) mid-late-logarithmic, (5) late-logarithmic, (6) early-stationary and (7) stationary (O/N) phase of growth. Blots were hybridized with probes specific to *luxS *and rRNA16S (acting as loading control). The estimated sizes of transcripts are indicated. The blots shown are representative of four independent experiments. Identical *luxS *expression profiles were observed in RDN02 (wild-type M19 strain) and RDN306 (isogenic *luxS*-deficient mutant). (B) Determination of the transcriptional start site of *luxS*. Left: Primer extension analysis using total RNA isolated from RDN29 wild-type cultures at lag (lag) and early-logarithmic (el) phase and the γ-^32^P-radiolabelled reverse primer oliRN250. As a reference ladder, a DNA sequencing reaction using plasmid pEC83 as template and reverse primer oliRN250 is shown (lanes TGCA). The complementary base (C*) of the transcriptional start site (G), located 20 nucleotides upstream of the translational start codon (ATG), is indicated. The picture shown is representative of three independent experiments. Right: Sequence of the 5' part of the *luxS *locus. The putative -35 and -10 promoter regions and +1 transcriptional start site are represented in bold. The putative ribosomal binding site (RBS) and translational start codon (ATG) are underlined. The arrow indicates the direction of primer oliRN250, which was used for primer extension. An inverted repeat sequence located 97 bp downstream of the translation stop codon could serve as a rho-independent transcriptional terminator (not shown).

**Figure 2:**
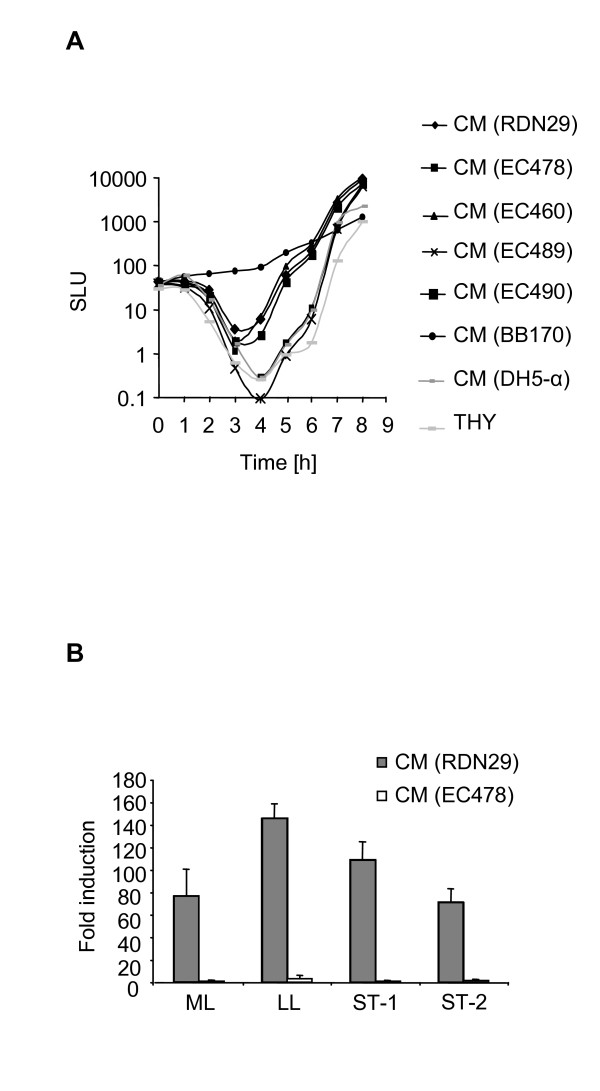
**Induction of luminescence in *V. harveyi *by CM from *S. pyogenes*.** (A) Induction of luminescence in *V. harveyi *reporter strain BB170 by CM of wild-type and *luxS*-deficient mutants. RDN29: wild-type M1 strain, EC460: RDN29 (pEC82), EC478: isogenic *luxS*-deficient mutant, EC489: EC478 (pEC82), EC490: EC478 complemented with pEC83. THY medium and CM of DH5-α (AI-2 deficient) were used as negative controls; CM of BB170 was used as positive control. CM were prepared from cultures at late-logarithmic phase of growth. The luminescence values expressed in specific light units (SLU = RLU (relative light units)/OD_620 nm_) (y axis) are plotted versus time (h) (x axis). Data shown are representative of three independent experiments. Similar data were obtained with CM of the RDN02 (M19 serotype) derivative strains. (B) Fold induction of *V. harveyi *BB170 luminescence by CM of RDN29 and EC478. CM was prepared from cultures at mid-logarithmic (ML), late-logarithmic (LL), early-stationary (ST-1) and stationary phase (ST-2) of growth. Fold induction corresponds to the SLU values obtained with the CM of the analyzed strains versus those obtained with the CM of the negative control THY. Fold induction was calculated after ~4 h of induction when the cell density begins to rise. Induction of luminescence with late-logarithmic CM from the *luxS*-deficient mutant was ~60 fold lower compared to CM from the parent wild-type strain. The average values and standard deviations of three independent experiments are shown. Similar data were obtained with CM of the RDN02 derivative strains.

First, we investigated *luxS *expression at the transcriptional level. Northern blot analysis of RNA extracts from wild-type strains revealed a monocistronic *luxS*-specific transcript of approximately 650 bases in size (Fig. 1A). In both strains, the *luxS *transcript levels were abundant from lag to early-logarithmic phase, after which gradual decrease of the signal intensity towards stationary phase was observed (Fig. 1A). Primer extension analysis further identified the transcription initiation site (+1) as a guanine located 20 nucleotides upstream of the translation start codon (ATG) (Fig. 1B). Then, we investigated whether the growth phase-dependent expression of *luxS *transcripts correlated with production of AI-2 like activity. Analysis of conditioned media (CM) from the *luxS*-deficient mutants demonstrated a loss of AI-2 like activity production in a bioluminescence reporter assay described earlier [9]. In contrast, CM from the wild-type parental strains induced luminescence in the AI-1 sensor defective reporter *V. harveyi *strain BB170 with AI-2 like activity peaking at late-logarithmic phase (Fig. 2A, B). CM prepared from the complemented *luxS*-deficient strains restored luminescence production in the reporter *V. harveyi *strain to a level comparable to that of wild-type CM (Fig. 2A). Furthermore, we showed that the *S. pyogenes luxS *gene complemented successfully the lack of AI-2 production in *E. coli *DH5-α (data not shown), thus confirming that the *luxS *homologue in both M1 and M19 strains is required for production of AI-2 like activity. We then addressed the question whether CM with AI-2 like activity has an auto-regulatory effect on *luxS *expression in *S. pyogenes*. CM from wild-type and *luxS*-deficient cultures grown to different phases was added to early-logarithmic wild-type cultures. No significant differences in the intensity of the *luxS *transcript signal were observed by Northern blot analysis when comparing induction with CM from both wild-type and mutant strains and control media (data not shown). Taken together, our data demonstrate that *luxS *transcription and production of AI-2 like activity are shifted out of phase. AI-2 like activity peaked at a time point – late-logarithmic phase – when *luxS *transcript levels rapidly decreased. Importantly, an auto-regulatory function of AI-2 like activity containing CM on *luxS *expression could not be observed in GAS.

### Role of *luxS *in growth and metabolism

Growth rates and yields, colony and chain morphology of wild-type and *luxS*-deficient strains in complex THY medium, THY medium supplemented with 10% fetal bovine serum, C-medium and CDM were similar (data not shown). In addition, wild-type and *luxS*-deficient strains did not significantly differ in their ability to form primary adhesion to uncoated polystyrene surfaces (initial step in biofilm formation) when grown in THY or CDM (data not shown). To investigate whether inactivation of *luxS *and therefore disruption of the AMC would lead to a metabolic burden that might influence growth or fitness, we analyzed the growth rates of wild-type and *luxS-*deficient strains in sulfur-limited CDM (CDM-S1 and CDM-S2). Although overall growth rates and yields were significantly reduced in the restricted media compared to those in CDM, they were similar when wild-type strains were compared to the *luxS*-deficient mutants (data not shown). These results indicate that *luxS *does not have an essential AMC-related metabolic role in GAS.

**Figure 3:**
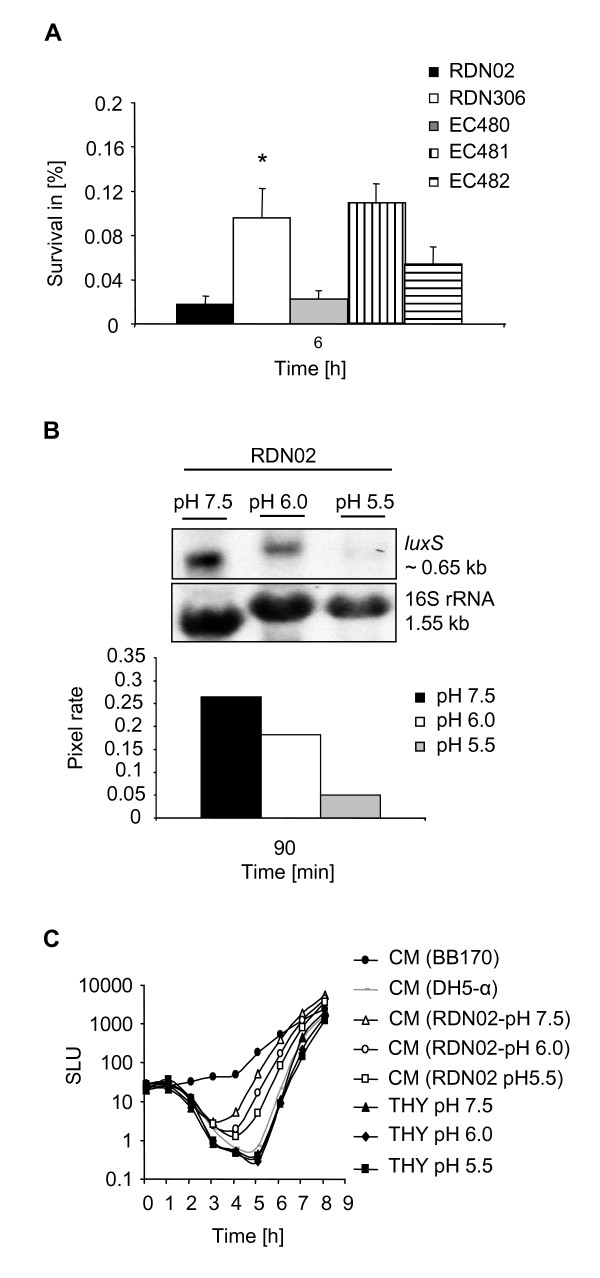
**(A) Acid tolerance assay.** The ability of RDN02 (wild-type M19 strain), RDN306 (isogenic *luxS*-deficient mutant), EC480 (RDN02 (pEC82)), EC481 (RDN306 (pEC82)) and EC482 (RDN306 (pEC83)) to survive an acid challenge (pH 4) is shown as survival in percent (y axis) at the 6 h time point. The average % survival values and standard deviations of three independent experiments are shown. The asterisk indicates that the difference in survival rates between the *luxS*-deficient mutant and the wild-type strain was statistically significant (*P *< 0.05). Similar data were obtained with the RDN29 (M1 type) derivative strains. (B) Effect of low pH on *luxS *expression. Northern blot analysis was performed with total RNA isolated from RDN02 cultures grown to early-logarithmic phase and then incubated for 90 min with THY medium at pH 5.5, 6.0 and 7.5. Blots were hybridized with probes specific to *luxS *and rRNA16S (acting as loading control). The estimated sizes of transcripts are indicated. Pixel rates of signals (pixel counts obtained with the *luxS *probe versus pixel counts obtained with the rRNA16S probe) are shown. The blot shown is representative of three independent experiments. Similar data were obtained with the RDN29 strain. (C) Induction of luminescence in *V. harveyi *reporter strain BB170 by CM of RDN02. CM was prepared from RDN02 cultures grown to early-logarithmic phase and then incubated for 90 min with THY medium at pH 5.5 (RDN02-pH 5.5), 6.0 (RDN02-pH 6.0) and 7.5 (RDN02-pH 7.5) for 90 min. THY medium at pH 7.5, 6.0 and 5.5 and CM of DH5-α (AI-2 deficient) were used as negative controls; CM of BB170 was used as positive control. The luminescence values expressed in specific light units (SLU = RLU (relative light units)/OD_620 nm_) (y axis) are plotted versus time (h) (x axis). Data shown are representative of three independent experiments. Similar data were obtained with CM of the RDN29 derivative strains.

Role of *luxS *in adaptation to host-induced stress conditions. Further, we investigated whether *luxS *is involved in the adaptation of *S. pyogenes *to stress conditions that can be encountered during infection. Oxidative, acid and salt stresses, which have been shown to interfere with virulence factor expression in GAS [28-30], were studied. Challenging wild-type and *luxS*-deficient strains with hydrogen peroxide (4 mM) and high salt concentrations (150 and 650 mM NaCl) did not induce any significant change in survival rate (data not shown). However, exposure of bacterial cultures to acidic conditions (pH 4) for 2, 4 and 6 h revealed a profound increase in acid tolerance in the *luxS*-deficient strains (Fig. 3A). Complementing the *luxS*-deficient strains with *luxS in trans *resulted in restoration of acid sensitivity to a wild-type level (Fig. 3A, shown only for the 6 h time point), clearly linking *luxS *to the observed phenotype. Importantly, we could show that exposing *S. pyogenes *cultures to low pH led to reduced expression of *luxS *(Fig. 3B), thus suggesting a correlation between reduced *luxS *expression and survival under acidic conditions. Accordingly, CM from these cultures showed reduced AI-2 like activity (Fig. 3C).

**Figure 4:**
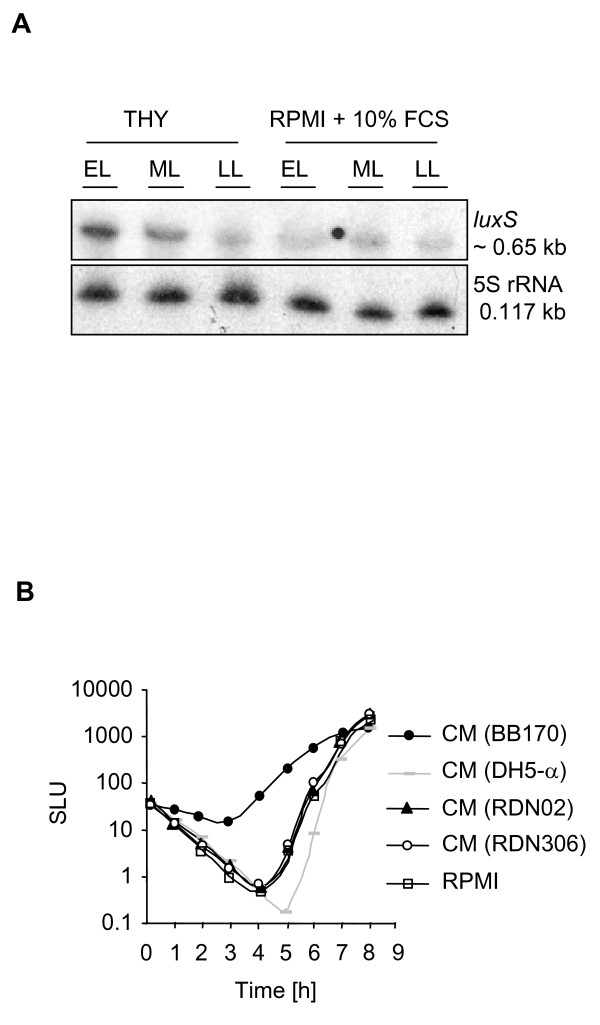
**(A) Analysis of *luxS *expression in RPMI medium.** Northern blot analysis of total RNA isolated from RDN02 (wild-type M19 strain) cultures at early-logarithmic (EL), mid-logarithmic (ML) and late-logarithmic (LL) phase of growth. Blots were hybridized with probes specific to *luxS *with 5S rRNA as loading control. The estimated sizes of transcripts are indicated. The blots shown are representative of three independent experiments. Identical *luxS *expression profiles were observed with RDN29 (wild-type M1 strain). (B) Induction of luminescence in *V. harveyi *reporter strain BB170 by CM of *S. pyogenes*. RDN02: M19 wild-type, RDN306: isogenic *luxS*-deficient mutant. RPMI medium and CM of DH5-α (AI-2 deficient) were used as negative controls; CM of BB170 was used as positive control. CM was prepared from cultures at late-logarithmic phase of growth in RPMI medium. The luminescence values expressed in specific light units (SLU = RLU (relative light units)/OD_620 nm_) (y axis) are plotted versus time (h) (x axis). Data shown are representative of three independent experiments. Similar data were obtained with CM of derivative strains of the RDN29 background.

Then, we analyzed the role of the *luxS*/AI-2 system in adaptation to an environment that resembles *in vivo *conditions by using RPMI medium (used for culturing eukaryotic cells) and 10% serum. We observed reduced *luxS *expression at the transcriptional level in wild-type cultures grown in RPMI medium compared to cultures grown in the nutrient rich THY medium (Fig. 4A). Furthermore, AI-2 activity in CM from wild-type cultures grown in RPMI was reduced to a level similar to CM from *luxS*-deficient cultures (Fig. 4B). During growth in RPMI, due to the presence of buffer salts and cultivation in the presence of CO_2_, no acidification occurs. Therefore, under this growth condition, regulation of *luxS *expression cannot be attributed to low pH. Since low concentration of iron in the RPMI medium could be responsible for the observed phenotype, we supplemented RPMI medium with iron and depleted THY medium from iron. In both cases, no change in *luxS *expression could be observed (data not shown). Our data thus indicate that under specific conditions encountered in the host (acidic conditions and *in vivo*-like environment), there is an advantage for GAS to lower the expression of *luxS *and the production of AI-2 like activity.

**Figure 5:**
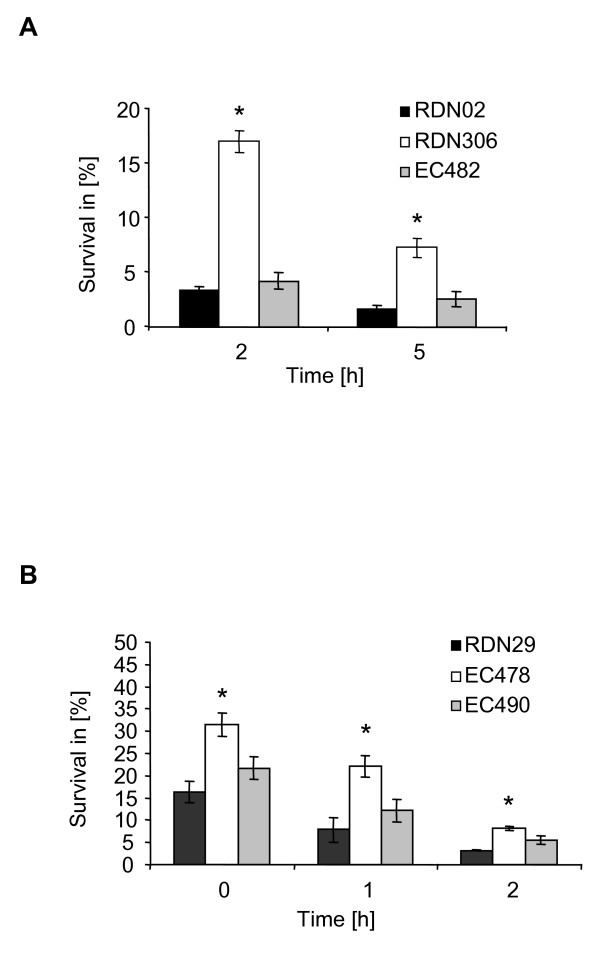
**Internalization assays of *S. pyogenes *in epithelial cells and macrophages.** RDN29 (wild-type strain), EC478 (isogenic *luxS*-deficient mutant) and EC490 (RDN29 (pEC85)); RDN02 (wild-type strain), RDN306 (isogenic *luxS*-deficient mutant) and EC482 (RDN29 (pEC85)). Bacterial cultures were used to infect (A) HEp-2 human pharyngeal epithelial cells and (B) RAW 264.7 macrophages in an antibiotic protection assay. For this, bacterial cultures were grown to mid-logarithmic (RAW 264.7) and late-logarithmic (HEp-2) phases, growth time points showing optimal internalization rates in RAW 264.7 and HEp-2, respectively. The percent intracellular invasion represents the average CFU/ml number of viable intracellular bacteria recovered at different times following addition of antibiotics versus the average CFU/ml number of inoculated bacteria × 100. The average values and standard deviations of three independent assays are shown. The asterisks indicate that the differences in survival rates between the *luxS*-deficient mutants and the wild-type strains were statistically significant (*P *< 0.01 for Hep-2 epithelial cells, *P *< 0.05 for RAW 264.7 macrophages). Similar data were obtained for both M1 and M19 serotypes.

Role of *luxS *in internalization and survival in human epithelial cells and macrophages. These results prompted us to determine whether a lack of *luxS *expression gives a beneficial advantage for GAS to survive in an intracellular environment. Interactions of wild-type and *luxS*-deficient strains of both serotypes M1 and M19 with monolayers of human pharyngeal epithelial cells were analyzed. No differences in the number of bacteria adhering to the epithelial cells (30 min, 1 h and 2 h after infection) were observed when comparing wild-type and mutant strains (data not shown). Using an antibiotic protection assay, the internalization and survival rates of the *luxS *mutant strains of both M1 and M19 serotypes were significantly higher (about 5-fold) at 2 h and 5 h after the addition of antibiotics compared to those of wild-type strains (Fig. 5A). Analyzing the number of bacteria that survived intracellularly in macrophages led to the same results. The *luxS *deficient mutants showed significantly higher internalization and survival rates (about 2.5-fold) in macrophages at 0 h, 1 h and 2 h after the addition of antibiotics compared to the wild-type parents (Fig. 5B). With both serotypes and in both epithelial and macrophage cells, complementing the mutant strains with the *luxS *allele *in trans *restored the intracellular survival rates to almost wild-type levels. Since the role of *luxS *in the AMC-related metabolic pathway seems limited, we analyzed the possibility of a signalling function of the *luxS*/AI-2 system in GAS survival in host cells. Antibiotic protection assays were performed with mixed cultures of wild-type and *luxS*-deficient cells (ratio 1:1) comparing with non-mixed wild-type and *luxS*-deficient cultures. The survival rates of the mixed culture in both eukaryotic cell types were almost identical to those of the wild-type strain (data not shown), thus suggesting a potential complementation effect of the *luxS*-deficient cells by the wild-type parent cells possibly by AI-2 production and signaling. In summary, the data indicate that lowering the expression of *luxS *provides GAS an advantage to survive the harsh conditions encountered in host cells.

### Virulence gene expression in *luxS *mutants

**Figure 6:**
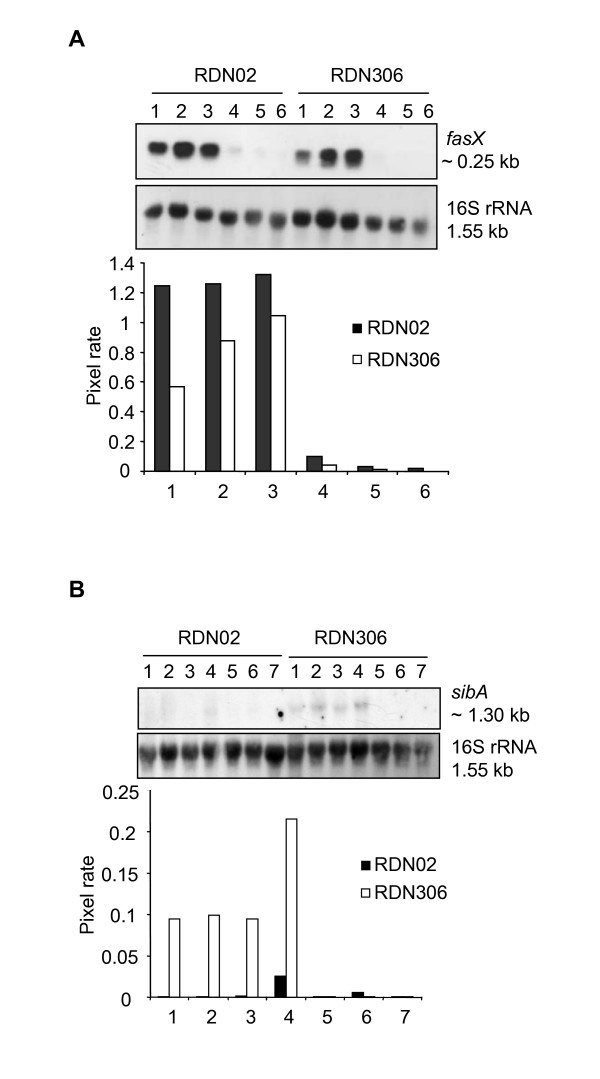
**Transcriptional expression of virulence-associated genes in RDN02 (wild-type M19 strain) and RDN306 (isogenic *luxS*-deficient mutant).** Northern blot analysis was performed with total RNA isolated from strains grown to (1) lag, (2) early-logarithmic, (3) mid-logarithmic, (4) mid-late-logarithmic, (5) late-logarithmic, (6) early-stationary and (7) stationary phase (O/N). Blots were hybridized with probes specific to *fasX *(A) and *sibA *(*psp*) (B) with a probe specific to 16S rRNA as loading control. The estimated sizes of transcripts are indicated. Pixel rates of signals (pixel counts obtained with probe of interest versus pixel counts obtained with the 16S rRNA probe) are shown for the different time points during growth. The blots shown are representative of three independent experiments.

We also studied whether *luxS *had regulatory effects on pathogenicity functions other than those already described (e.g. effect on cysteine protease, SLS and hyaluronic acid production) [26,27]. Expression of a number of genes encoding virulence factors and their regulators [16,21,28,31-36] was then studied by Northern blot analysis throughout the entire growth phase (Table 2). A consistent decrease in the *fasX *(encoding the effector molecule of the *fasBCA *regulatory system [21]) transcript and a consistent enhancement of the *sibA *(*psp*) (encoding a secreted immunoglobulin binding protein [32]) transcript were detected in the *luxS*-deficient RDN02 (M19) strain compared to the wild-type parent, however not in the M1 background (Fig. 6A, B). This finding does not only add *fasX *and *sibA *to the list of genes being influenced by *luxS *but underlines the fact that regulatory pathways linking the *luxS*/AI-2 pathway to virulence-associated gene expression can differ among different GAS strains or serotypes.

**Table 2:** Oligonucleotides used in this study

Oligo	^a^F/R	Sequence 5'-3'	^b^Specificity
oliRN110	F	CGCTGCGTAAAAGATACG	*aphIII*

oliRN111	R	AATGGAGTGTCTTCTTCC	

oliRN130	F	TTGAAATGACAAAAGAAGTT	*luxS*
oliRN129	R	AAAGAGGCTATGATCCTTAT	

OLEC287OLEC288	F	AGTTAAGTGACGATAGCCTAG	5S rRNA
	R	CTAAGCGACTACCTTATCTCA	

oliRN242	F	CGGTAACTAACCAGAAAGGG	16S rRNA
oliRN243	R	CGTTGTACCAACCATTGTAGC	

			Virulence genes
OLEC137	F	GACCTACTCAGGCAAATC	*fbp54*
OLEC138	R	CTTGTCTAAGGCAATCTC	
oliRN204oliRN203	F	TGGTAAAAATGCCTATACCC	*isp2*
	R	GACCTCGTGAAAACTTAGCC	
oliRN248	F	AGGAATAAATTGGTCCTCTT	*nga*
oliRN249	R	GGCAGTTTCAATAAACTCAT	
OLEC268	F	AAGAGATCGAAGAAAGTCTT	*scl*
OLEC269	R	TTTGGTTAGCTTCTTTGTCA	
oliRN202oliRN201	F	AGTTAAGGCACAAGAACAGG	*sibA *(*psp*)
oliRN246	R	TACTTGAGGCACTTTGAACC	
oliRN247	F	ACTAGGAGCTACACAACCAG	*sic*
oliRN263	R	TACCCTGTACCTAATGCTTC	
oliRN264	F	ACATCTCAAGAATTACTAGC	*ska*
oliRN279	R	TTGTTGTAGAGTAGTTTAGC	
oliRN50	F	ATGGAAAATATTCTGATATCTTAG	*slo*
oliRN47	R	CCCAAAGGATTTCATATTGAGC	
	F	CATAATTACAGTCACTGATT	*speC*
oliRN48	R	ATCGAAATGACTAAAGTTCTTCAT	
oliRN55	F	TTTTCAATGGTAGCTCTTG	*speF (mf)*
oliRN56	R	TTCTTGAGCTCTTTGTTCG	

			Stand-alone regulators
OLEC274	F	ATAGCCTAGAAACAGAATTA	*codY*
OLEC275	R	TTCTGAATAAGAAAGCGTAT	
oliRN170	F	TAAGTAAGTTGTTTACAAGTCAAC AGTGGAG	*mga*
oliRN136	R	AAAGGCGTAGATCAATTGG	
OLEC337	F	ATGACGCCTAATAAAGAAGA	*mtsR*
OLEC338	R	CTGTGACATAAAGTTGCTTA	
OLEC315	F	TGGACATTCATTCACATCAG	*perR *(*spf*)
OLEC316	R	ATCAGGTTGGTCTTTTGCTT	
oliRN73	F	AAATACTTGGAATCATCAATCG	*rofA*
oliRN74	R	TTTTCTTGAGCTAATGCAACCG	
OLEC278	F	TAGACAACTTGAATGTCAAT	*ropB*
OLEC279	R	AAGCTTTATCATACTCTTGT	
OLEC276	F	ACATTTTGCAACGGTATATT	*srv*
OLEC277	R	AATAGGGTCATTAAGTCATA	

			Two-component systems
oliRN175	F	CTGCTTGACTTAATGTTACC	*covR *(CovRS)
oliRN176	R	TTTGACAATAATTCTTCACG	
OLEC182	F	GAGCAATAACATTTTAGG	*fasX *(effector molecule, FasBCA)
OLEC183	R	TTACAATCAGCTGATGTG	
OLEC270	F	ATTCGAAAGACATCAGATGT	*Irr *(Ihk/Irr)
OLEC271	R	CATATGTCATCCATCAATTG	
OLEC339	F	GATATCATGATGCCGATTAA	*sptR *(SptRS)
OLEC340	R	TAGCTAGTATCTTTATCACC	
OLEC280	F	TACTTATTGTGGATGATGAA	*vicR *(VicRK)
OLEC281	R	AATCATATCCCCAAACAATT	

oliRN23	F	AATTGTGAATTCTATCATAATTGTGG (*Eco*RI)	Cassette *aphIII *for *luxS*- deficient mutants
oliRN24	R	TAAATCAAGCTTCTAAAACAATTCATCCAG (*Hin*dIII)	

oliRN20	F	ACTAGTGAATTCTCAAACATAACAATCC (*Eco*RI)	Fragment *luxS*down for *luxS*- deficient mutants
oliRN19	R	TTCTGAGGATCCCCACCATCCAGCC (*Bam*HI)	

oliRN22	F	AATCAAGCATGCTTACTTGGAAAAGAACCCAACC (*Sph*I)	Fragment *luxS*up for *luxS*- deficient mutants
oliRN21	R	AAGTGGAAGCTTGTGGAGGAGAAAAGGC (*Hin*dIII)	

oliRN267	F	CATCGCTGCGCCTCTTGCTAC	Confirmation of the *luxS*- deficient mutants
oliRN268	R	GGGTCATTCCAGAACCACAGA	
oliRN70	F	GCTATTTTTTGACTTACTGGG	
oliRN69	R	TCCGTATCTTTTACGCAGCGG	

oliRN205	F	GTCAATGGATCCCCAGCTCTATTGCACC (*Bam*HI)	P*luxS*-*luxS*-TT insert for *luxS *complementation plasmid pEC83
oliRN280	R	TATCTAGAGCTCTAGATTACTGAGAAAATC (*Sac*I)	

oliRN250	R	ATTCTTCAGAAATAAGACG	Primer extension

^a ^F, forward; R, reverse. Underlined sequences indicate restriction sites.

^b ^*fbp54 *(fibronectin-binding protein), *isp2 *(immunogenic secreted protein homologue), *nga *(NAD-glycohydrolase), *scl *(streptococcal collagen-like protein), *sibA *(secreted immunoglobulin binding protein), *sic *(streptococcal inhibitor of complement), *ska *(streptokinase), *slo *(streptolysin O), *speC *(streptococcal pyrogenic exotoxin C), *speF *(*mf*) (mitogenic factor).

## Discussion

Among the quorum sensing systems described to date, the *luxS*/AI-2 pathway has been shown to be involved in the regulation of virulence in a number of gram-negative and gram-positive bacteria [1,5,11,12]. In this study, we analyzed the expression of the streptococcal *luxS*/AI-2 system, its possible role in the AMC metabolic pathway and its function in adaptation to diverse host-induced stress conditions in two GAS clinical isolates of serotypes M1 and M19.

Expression analysis of *luxS *indicated that the *luxS *transcript is monocistronic (644 bases) in GAS serotypes M1 and M19. In a previous report, which was based on sequence analysis, the *luxS *transcript in GAS (M6 strain) was predicted to be polycistronic as a distal member of the fatty acid metabolism operon [26]. In our study, Northern blot analysis of *luxS *expression did not reveal any additional transcript of higher size. Furthermore, we showed that the monocistronic transcript is expressed in a growth phase-dependent manner, a finding already reported for bacterial species like *Streptococcus bovis*, whereas transcription of *luxS *in species like *Salmonella typhimurium *and *Vibrio fischeri *has been shown to be constitutive [37-39]. Accordingly, production of AI-2 like activity occurred in a temporal fashion in the serotypes analyzed, however, shifted out of phase compared to *luxS *expression. AI-2 like activity peaked at late-logarithmic phase when the *luxS *transcript had already declined being just above the detectable expression level. We also investigated the possibility of an auto-regulation mechanism of *luxS *expression in GAS. Induction experiments by Northern blot analysis failed to show a regulation of *luxS *expression by AI-2 dependent CM. These findings are in accordance with previous work in *S. typhimurium*, where AI-2 production and *luxS *transcription were not in phase either, also suggesting that *luxS *expression was not regulated by AI-2 [38].

Despite intensive research on *luxS *and AI-2 during the last years, a clear separation of the possible metabolic function of AI-2 from its possible signaling activity could not be achieved. A recent comparative genomic and phylogenetic analysis of synthesis and signal transduction pathways showed that *S. pyogenes *contains the two-step enzymatic pathway catalyzed by the Pfs and LuxS enzymes to detoxify SAH leading to AI-2 production [13,40]. Interestingly, in *S. pyogenes *the AMC is incomplete as it lacks the homocysteine methyltransferases (MetE or MetH) that convert the SAH-derived metabolic product homocysteine to methionine. Nevertheless, *S. pyogenes *encodes the SAM synthetase ortholog (MetK) that permits conversion of methionine to SAM [40]. Therefore, either GAS cannot recycle homocysteine to methionine (as it has been described in *Borrelia burgdorferi *[41]) or recycling occurs through other types of methyltransferases. In the former case of a *de novo *methionine synthesis defect, bacterial growth would exclusively depend on sulfur provided from external sources. In our study, decreasing media concentrations of cysteine and methionine reduced to a similar extent bacterial growth and yields in both wild-type and *luxS*-deficient strains. These data indicate that GAS, as a poly-auxotrophic organism, is unable to recycle homocysteine to methionine. Moreover, we showed that inactivation of *luxS *does not lead to a metabolic burden that would influence growth or fitness. Although AI-2 can certainly be considered as a metabolite, the viability and lack of growth defects of the investigated *luxS*-deficient mutants in various media including minimal media depleted of sulfur sources argues against an essential role of *luxS *in AMC-related metabolism.

The previously described regulatory role of *luxS *in the production of virulence factors in GAS [26,27] prompted us to investigate the role of the *luxS*/AI-2 system in the adaptation to host-induced stress conditions. During an on-going infection, bacteria often have to face challenging conditions in particular niches of the host, including changes in pH, and therefore are forced to develop quickly an adaptive response, which requires fine-tuning of pathogenicity gene expression. Although we could not identify a possible involvement of the GAS *luxS *system in adaptation to oxidative and salt stress, we showed an increased survival of *S. pyogenes *under acidic conditions when the *luxS*/AI-2 system was down-regulated. This is in accordance with *luxS *expression and AI-2 production being significantly lowered when GAS cells were grown in low pH conditions. Interestingly, previous studies in *S. mutans *showed that acid sensitivity was enhanced in *luxS *deficient mutants [42,43] and that *luxS *expression was increased at low pH [44,45]. These reverse effects of *luxS *on tolerance to acidic conditions in *S. pyogenes *and *S. mutans *need to be considered in regard to their different living habitats and pathogenesis. Thus, our data reveal a link between the *S. pyogenes luxS*/AI-2 system and pathways involved in adaptation of the organism to stress [46,47]. In addition, we also observed a reduction of *luxS *expression and AI-2 like activity in GAS cells grown in serum enriched RPMI, a medium with a composition similar to that of human plasma. In a previous study, Marouni et al. reported that in an M6 serotype, the survival of a *luxS*-deficient mutant in epithelial cells at 4 h after infection was higher compared to the wild-type parent [27]. Here we analyzed further the role of *luxS *in interaction of *S. pyogenes *with host cells. No differences in adhesion rates to human pharyngeal epithelial cells were observed when comparing wild-type and *luxS*-deficient mutants in both M1 and M19 serotypes. However, the *luxS*-deficient mutants had a significant advantage to survive intracellularly in both epithelial cells (over a period of 7 h after infection) and macrophages (over a period of 3 h after infection). Taken together, we show that low level of *luxS *and AI-2 expression seems to provide a competitive advantage for GAS survival under specific conditions encountered during infection.

With *luxS *being linked to stress and adaptation to the host, we were interested in investigating additional effects of *luxS *on virulence factor expression in GAS. In an M6 serotype, *luxS *was shown to regulate streptolysin S (SLS) expression at the transcriptional level and SpeB cysteine protease activity [26]. In an M3 serotype, *luxS *was reported to regulate expression of SpeB and M protein at the transcriptional level and hyaluronic acid capsule at the post-transcriptional level [27]. In our report, we show that *luxS *had a positive effect on *fasX *expression and a negative effect on *sibA *(*psp*) expression at the transcriptional level. *fasX *is a small RNA molecule, effector of the *fasBCA *operon, which has regulatory functions on virulence factor expression. *fasX *was also suggested to be involved in local tissue destruction and general bacterial aggressiveness towards host cells [21,48]. *sibA *(*psp*) is a virulence gene encoding a secreted immunoglobulin binding protein [32]. Remarkably, the above described regulatory effects were only observed in the M19 strain, thus suggesting a strain or serotype dependent effect of *luxS *on the expression of these two targets. The strain- or serotype-dependent effect of *luxS *observed in this study emphasizes differences in regulatory pathways among different GAS isolates. Along these lines, previous reports demonstrated that mutations in GAS regulators might alter disease progression [49,50]. This is well illustrated with the two-component regulatory system CovRS where mutations in the sensor encoding gene *covS *have been shown to correlate with human disease severity [51,52]. Mutations in this gene can occur under selective pressure encountered in the host and can generate hypervirulent GAS variants with increased risk of systemic dissemination [51,52]. In the case of *luxS*, strain specificity manifestation of virulence-associated gene regulation by *luxS *has been reported previously in *Neisseria meningitidis *and *Serratia marcescens *[53,54]. Additionally, Marouni et al. attributed the dissimilar results of the effect of *luxS *on bacterial growth and the level of SpeB regulation in GAS to a possible strain-specific effect [27]. The finding that *luxS *can affect the expression of *fasX *RNA also provides an additional evidence for the notion that small RNAs have the ability to integrate cell density signals together with other environmental stimuli to affect gene expression [55].

## Conclusion

Our data together with previous reports suggest a complex role of the *luxS*/AI-2 system in *S. pyogenes*. Here, we showed that expression of both *luxS *and AI-2 occurs in a temporal fashion but AI-2 like activity does not seem to have a regulatory effect on *luxS *expression. Analysis of the role of *luxS *in metabolism demonstrated a limited role of *luxS *in the AMC-related metabolism. However, studying the possible function of the *luxS*/AI-2 system in adaptation to stress revealed that a down-regulation of *luxS *expression provides an advantage for *S. pyogenes *to tolerate acidic conditions and grow in a host environment-mimicking medium. Accordingly, there was an increased ability of the *luxS*-deficient mutants to survive intracellularly in both epithelial cells and macrophages. Altogether, our data suggest an important function for the *luxS*/AI-2 system in survival and growth of GAS under conditions that are relevant during infections. Based on the data outlined in this article, it is tempting to speculate that in GAS, *luxS *is not exclusively a key part of a detoxification pathway but rather modulation of *luxS *expression levels would allow adjusting bacterial fitness in response to changing host conditions. Finally, our study revealed two novel virulence-associated targets of *luxS *in *S. pyogenes*: the regulator *fasX *RNA and the virulence gene *sibA*.

## Methods

### Bacterial strains and growth conditions

Bacterial strains and plasmids used in this study are listed in Table 1. *S. pyogenes *was routinely cultured in Todd Hewitt Broth (THB, Bacto, Becton Dickinson) supplemented with 0.2% yeast extract (Oxoid) (THY) without agitation or on trypticase soy agar supplemented with 3% sheep blood (TSA, BBL, Becton Dickinson). C-medium (peptide rich and carbohydrate poor), THY or RPMI medium supplemented with 10% foetal bovine serum (Gibco), metal ion-restricted medium, chemically defined medium (CDM) and sulfur-restricted CDM (CDM-S) were used in specific experiments [56,57]. For the eukaryotic-like environment, RPMI 1640 (PAA) was supplemented with 10% foetal bovine serum (Gibco) or 10% foetal bovine serum and 50 μM FeCl_3_. For metal-ion restricted medium, THY was treated overnight with 30 g of chelating resin Chelex 100 (Sigma)/liter, supplemented with 1 mM MgCl_2 _or 1 mM MgCl_2_and 1 mM FeCl_3_. Sulfur-restricted CDM (CDM-S) consisted of CDM with sulfur containing salts replaced with their chloride equivalents [58]. CDM contained L-methionine (670 μM), L-cysteine (4,125 μM) and L-cystine (208 μM). In sulfur-limiting conditions, L-cystine was eliminated and only L-cysteine (10 μM or 350 μM) and L-methionine (1 μM or 100 μM) were provided as sulfur source. All *S. pyogenes *cultures were incubated at 37°C in an atmosphere supplemented with 5% CO_2_. *Escherichia coli *was grown aerobically at 37°C in Luria-Bertani (LB) medium either in liquid with shaking or on agar plates. *V. harveyi *was cultured aerobically at 28°C in an Autoinducer Bioassay (AB) medium [9]. Transformation of *E. coli *and *S. pyogenes *was performed as previously described [59,60]. Whenever required, suitable antibiotics were added to the medium to the following final concentrations: erythromycin 300 μg/ml for *E. coli *and 3 μg/ml for *S. pyogenes*; kanamycin 25 μg/ml for *E. coli *and 300 μg/ml for *S. pyogenes*. Bacterial cell growth was monitored periodically by measuring the optical density of culture aliquots at 620 nm using a microplate reader (SLT Spectra Reader).

### DNA manipulations

DNA manipulations including DNA preparation, amplification, digestion, ligation, purification, agarose gel electrophoresis and Southern blot analysis were performed according to standard techniques [60]. Synthetic oligonucleotides used as primers in this study (Table 2) were supplied by VBC-Biotech Services GmbH. Sequencing reactions were performed at VBC-Biotech Services GmbH.

### Construction of the *luxS*-deficient mutants

Replacement of the *luxS *coding sequence with a kanamycin resistance cassette, *aphIII *[61], was performed selecting for a double cross-over event. For this purpose, a 1209 bp fragment upstream of *luxS *(*luxS*-up) and a 1107 bp fragment downstream of *luxS *(*luxS*-down) were amplified using wild-type genomic DNA as template and primers (containing flanking restriction sites) oliRN22/oliRN21 and oliRN20/oliRN19, respectively. The *aphIII *cassette was amplified using plasmid pAT21 as template and primers oliRN23/oliRN24. After digestion with the respective restriction enzymes, the three fragments were ligated and cloned into pUC19 (suicide vector for *S. pyogenes*). The resulting plasmid pEC131 was purified, linearized with the restriction enzyme *Sca*I (which cuts within the ampicillin resistance cassette) and used to transform electro-competent wild-type *S. pyogenes*. Kanamycin resistant clones were selected and the correct replacement event was checked by PCR analysis using combinations of primers oliRN267 and oliRN268 derived from flanking regions upstream and downstream of the *luxS*-up and *luxS*-down fragments and primers oliRN69 and oliRN70 specific to the *aphIII *cassette. Southern blot analysis was done to further confirm that the recombination events had not affected the DNA regions located upstream and downstream of *luxS*. The *S. pyogenes *mutants were grown in liquid medium without antibiotic unless otherwise specified.

### Construction of plasmids for complementation studies

Plasmid pEC82 contains *repDEG*-pAMβ1 (the origin of replication of pAMβ1), *ermAM *(an erythromycin resistance gene with its own promoter and transcriptional terminator), ColE1 (a pUC19-based ColE1 origin of replication for *E. coli*) and an expanded MCS (multiple cloning site). To create the pEC83 *luxS*-complementation vector, a 1152 bp large DNA fragment (P*luxS*-*luxS*-TT) containing the *luxS *coding sequence, its putative promoter region and putative transcriptional terminator, was amplified from wild-type genomic DNA using primers (containing flanking restriction sites) oliRN205/oliRN280 and cloned into pEC82. Plasmids pEC82 and pEC83 were introduced in competent *E. coli *and *S. pyogenes *strains selecting for erythromycin resistant clones.

### RNA analysis

Total RNA was prepared from culture samples harvested at different time points during growth and further processed for normalization and Northern blot analysis as previously described [62] with minor modifications. Specific α-^32^P-dATP labeled DNA probes corresponding to internal fragments of genes were created by amplification of wild-type genomic DNA using primers described in Table 2. Primer extension analysis of the *luxS *transcript and DNA sequencing reactions were carried out according to standard protocols [60].

### Phenotypical studies

Assays for bioluminescence, acid killing, hydrogen peroxide killing, salt stress and biofilm formation were performed as described previously [9,28,29,43,63] with some minor modifications. For the acid tolerance assay, mid-logarithmic phase bacterial cultures were harvested by centrifugation and washed once with 0.1 M glycine buffer (pH 7.0). Culture aliquots were removed for CFU determination and the remaining cultures were subjected to killing by incubating the cells in 0.1 M glycine buffer (pH 4) for 6 h. The number of viable bacteria was determined by plating appropriate dilutions in triplicate on TSA media. For each individual experiment, the ability of strains to survive the acid challenge was reported as survival in percent, defined by the ratio of average CFU/ml (triplicate measurements) recovered after challenge versus the average number of CFU/ml (triplicate measurements) present immediately before challenge × 100.

### Preparation of conditioned medium for induction assays

Conditioned medium (CM) was prepared from bacterial cultures grown in THY buffered with 0.1 M HEPES (Sigma) (pH 7.5) to early-, mid- and late-logarithmic phase. Culture supernatants were separated from bacterial pellets by centrifugation at 9,500 × g for 30 min at 4°C, filter sterilized (0.45 μm) and stored at -20°C. For induction assays, early-logarithmic wild-type cultures were induced for 60 and 90 min with THY adjusted to pH 7.5, 6.0 and 5.0 or with CM of wild-type and *luxS*-deficient cultures from early-, mid- and late-logarithmic growth phase and then subjected to Northern blot analysis.

Bacterial adhesion, internalization and survival assays in host cells. HEp-2 cells and RAW 264.7 mouse macrophages were maintained in RPMI medium (HEp-2 cells) or Dulbecco's modified Eagle's medium (D-MEM, Gibco) (RAW 264.7 cells) supplemented with 10% foetal bovine serum (Gibco), penicillin (100 μg/ml) and streptomycin (100 μg/ml), and grown at 37°C in an atmosphere containing 5% CO_2_. Prior the infection assay, the cells were cultured overnight to semi-confluency (1.5 × 10^5 ^cells per well for HEp-2 cells and 2 × 10^5 ^cells per well for RAW264.7 cells) in 24-well tissue culture plates containing medium without antibiotic. GAS strains were grown to the same OD_620 nm _corresponding to mid-logarithmic (RAW 264.7) or late-logarithmic (HEp-2) phase, washed with PBS, suspended in fresh cell culture medium and added to the semi-confluent host cell monolayers at a multiplicity of infection (MOI) of 5:1 (HEp-2) or 100:1 (RAW 264.7) in triplicate. Times of incubation were 30 min, 1 h and 2 h (adhesion, HEp-2 cells), 2 h (internalization, HEp-2 cells) and 30 min (RAW 264.7 cells). For adhesion assays (HEp-2 cells), infected monolayers were washed extensively with PBS before lysing. For internalization assays (RAW 264.7 cells), extracellularly adhered bacteria were killed by incubation with fresh medium containing either 100 μg/ml gentamicin and 5 μg/ml penicillin (HEp-2 cells) or 60 μg/ml penicillin (RAW 264.7 cells). At indicated time points, the infected monolayers were washed extensively with PBS to remove the antibiotics. Cells were then lysed with chilled sterile distilled water. The number of viable intracellular bacteria released from the lysed cells was determined by plating appropriate dilutions of the lysates on TSA-blood plates in triplicates followed by 24 h incubation at 37°C. The number of bacteria that survived intracellularly was calculated using the following equation: average number of bacteria recovered (cfu/ml) per well (triplicate) at designated time point/inoculated number of bacteria (cfu/ml)) × 100. Average values were plotted.

## Authors' contributions

MS, RPJ, ZAP, CH and DZ carried out the experimental work. MS, RPJ and CH participated in the design of the study. MS helped to draft and critically revised the manuscript. MS designed the figures. EC conceived the study and wrote the manuscript. All authors read and approved the final manuscript.
